# Entwicklung der Epidemiologie antibiotikaresistenter Erreger in der Humanmedizin und deren Bedeutung für das Gesundheitssystem

**DOI:** 10.1007/s00103-026-04234-6

**Published:** 2026-04-27

**Authors:** Tim Eckmanns, Felix Reichert, Marc Schneider, Marcel Feig, Ines Noll, Alexandra Hoffmann

**Affiliations:** Abteilung für Infektionsepidemiologie, Robert Koch-Institut, Seestr. 10, 13353 Berlin, Deutschland

**Keywords:** Antimikrobielle Resistenz, Antibiotika-Resistenz-Surveillance (ARS), EARS-Net, IHME/MICROBE, DART-Ziele, Antimicrobial resistance, Antibiotic resistance surveillance (ARS), EARS network, IHME/MICROBE Project, DART aims

## Abstract

Die Weltgesundheitsorganisation (WHO) stuft Antibiotikaresistenz (AMR) als eine der 10 größten globalen Bedrohungen ein, die zu erheblicher Morbidität, Mortalität und wirtschaftlicher Belastung führt. Auch in Deutschland sind die Entwicklungen der Resistenzsituation teilweise kritisch. Die Resistenzanteile der wichtigsten Erreger-Wirkstoff-Kombinationen werden mithilfe von Daten der Antibiotika-Resistenz-Surveillance (ARS) berechnet. Für 4 Erreger-Wirkstoff-Kombinationen wurden im Aktionsplan der Deutschen Antibiotika-Resistenzstrategie (DART 2030) Reduktionsziele für Inzidenzen (Blutstrominfektionen pro 100.000 Einwohnende) für das Jahr 2030 festgelegt. Während für die grampositiven Erreger Vancomycin-resistente *Enterococcus faecium* (VRE) und Methicillin-resistente *Staphylococcus aureus* (MRSA) die Inzidenzen bereits im Zielbereich liegen, ist dies für *Escherichia coli* mit Resistenz gegen Cephalosporine der 3. Generation und *Klebsiella pneumoniae* mit Resistenz gegen Carbapeneme nicht der Fall und die Inzidenzen steigen an. Im EU-Vergleich liegt Deutschland bei den Inzidenzen im Mittelfeld.

Neben Resistenzanteilen und Inzidenzen gibt das MICROBE-Projekt (Measuring Infectious Causes and Resistance Outcomes for Burden Estimation) absolute Anzahlen von Todesfällen durch Krankheitsepisoden mit resistenten Erregern an. Es sterben pro Jahr über 40.000 Menschen in Deutschland an Infektionen mit antibiotikaresistenten Erregern, bei fast 10.000 dieser Fälle ist die Resistenz der Erreger die Ursache. Damit sterben an solchen Infektionen deutlich mehr Menschen in Deutschland als an anderen Infektionskrankheiten.

Die Auswirkungen auf das Gesundheitssystem sind erheblich, so führt die zunehmende Antibiotikaresistenz unter anderem zu einer erhöhten Krankheitslast bei hämatoonkologischen Krankheiten und Herausforderungen bei der perioperativen Antibiotikaprophylaxe.

**Zusatzmaterial online:**

Zusätzliche Informationen sind in der Online-Version dieses Artikels (10.1007/s00103-026-04234-6) enthalten.

## Abstract (en)

The World Health Organization (WHO) has ranked antibiotic resistance as one of the 10 biggest global threats, leading to significant morbidity, mortality, and economic burden. In Germany the developing resistance situation is critical. The resistance data of the most relevant drug–bug combinations are calculated with data from the German Antibiotic Resistance Surveillance (ARS) system. For four of these drug–bug combinations, incidence reduction targets for 2030 (bloodstream infections per 100,000 inhabitants) have been outlined in the German Antimicrobial Resistance Strategy (DART 2030) action plan. The incidence for the gram-positive vancomycin-resistant *Enterococcus* (VRE) and methicillin-resistant *Staphylococcus aureus* (MRSA) are currently within the target range. However, this is not the case for *Escherichia coli* with resistance to third generation Cephalosporins and Carbapenem-resistant *Klebsiella pneumoniae*, both of which are increasing in incidence. In an EU comparison, the incidence of AMR in Germany ranks in the middle.

In addition to the resistance data and incidence, the Measuring Infectious Causes and Resistance Outcomes for Burden Estimation (MICROBE) project presents the absolute numbers of mortalities from infections with antimicrobial-resistant pathogens. In Germany, 40,000 deaths per year are associated with antibiotic-resistant infections, and in almost 10,000 of these cases the death was attributable to the antibiotic-resistant pathogen. This means that more people in Germany are dying from antibiotic-resistant infections than other infectious diseases.

The effects on the healthcare system are substantial; the increasing antibiotic resistance in particular leads to greater burden of disease in haemato-oncological diseases and poses challenges to effective perioperative antibiotic prophylaxis.

## Einleitung

Die Weltgesundheitsorganisation (WHO) stuft Antibiotikaresistenz (AMR) als eine der 10 größten globalen Bedrohungen ein, die zu erheblicher Morbidität, Mortalität und wirtschaftlicher Belastung führt. Trotzdem wird dieses Problem in den öffentlichen Gesundheitsdiskussionen weltweit unzureichend behandelt. Das liegt auch an der mangelnden Kenntnis des Ausmaßes und der komplexen Thematik. Es handelt sich nicht um eine Krankheit oder einen Erreger, sondern um viele verschiedene Erreger wie *Staphylococcus (S.) aureus, Escherichia (E.) coli* und *Pseudomonas (P.) aeruginosa*, die einigen noch bekannt sein mögen. Hinzu kommen ausschließlich bei mikrobiologischen Expert:innen bekannte Erreger wie *Enterobacter hormaechei*, dessen Carbapenem-resistente Variante jedes Jahr in Deutschland über 100-mal nachgewiesen wird und im Jahr 2025 einen Ausbruch in mehreren Ländern der EU verursacht hat. Diese vielen unterschiedlichen Erreger wiederum können jeweils verschiedene Krankheiten verursachen.

Die Kenntnisse der Resistenzsituation nutzen den Verschreibenden von Antibiotika bei der Wahl des Antibiotikums in dieser komplexen Situation. Für Nichtfachleute ist die Beurteilung herausfordernd: Mal ist ein Resistenzanteil von 5 % als sehr hoch zu werten, z. B. bei Carbapenem-resistenten *Klebsiella (K.) pneumoniae*, mal gilt der gleiche Anteil als sehr niedrig, z. B. bei Vancomycin-resistenten *Enterococcus (E.) faecium* (VRE).

Der Resistenzanteil gibt den Anteil resistenter Erreger von allen gegen diese Substanz getesteten Erregern an. Neben dieser Angabe werden in den letzten Jahren zunehmend Maßzahlen wie die absolute Anzahl von Patient:innen-Episoden, d. h. Ereignissen wie Blutstrominfektionen mit resistenten Erregern bei einer Patient:in, und Inzidenzen, d. h. die Anzahl resistenter Erreger pro 100.000 Einwohnende, verwendet. So wird das Problemfeld der Antibiotikaresistenz verständlich dargestellt, es ist eine bessere Vergleichbarkeit mit anderen Krankheiten gegeben und es besteht die Möglichkeit, die Krankheitslast durch verschiedene resistente Erreger aufzuaddieren.

Erstmalig wurden im Jahr 2023 durch die EU-Ratsempfehlung sowie in der Deutschen Antibiotika-Resistenzstrategie (DART 2030) Ziele für das Jahr 2030 definiert [1, 2]. So sollen je nach Ausgangswert im Land prozentuale Reduktionen der Inzidenzen bestimmter Blutstrominfektionen erreicht werden, und zwar für *E. coli* mit Resistenz gegen Cephalosporine der 3. Generation, *K. pneumoniae* mit Resistenz gegen Carbapeneme und Methicillin-resistente *S. aureus* (MRSA). Die EU-Ziele wurden in dem die DART 2030 ergänzenden 1. Aktionsplan übernommen sowie ein weiteres Reduktionsziel für VRE hinzugefügt [3].

Im Folgenden werden unterschiedliche Maßzahlen zur Antibiotikaresistenz in Deutschland aus verschiedenen Datenquellen vorgestellt. Diese werden ins Verhältnis zu entsprechenden Zahlen anderer Länder der EU und des Europäischen Wirtschaftsraums (EWR) sowie zu Erregern anderer Infektionskrankheiten in Deutschland gesetzt. Ziel ist es, aktuelle Daten zur Antibiotikaresistenz und deren Stand im Vergleich zu den DART-Zielen in Deutschland darzustellen.

## Aktuelle Krankheitslast durch Antibiotikaresistenz

Antibiotikaresistenz stellt eine erhebliche globale Bedrohung für die menschliche Gesundheit und die Zukunft der modernen Medizin dar. Resistenzen von Bakterien gegenüber Antibiotika sind ein natürliches Phänomen. Wahrscheinlich seitdem Bakterien und andere Mikroorganismen existieren, haben diese Mikroorganismen Substanzen gegen Bakterien und Bakterien wiederum Resistenzen gegen diese Substanzen entwickelt [4]. Solange dies natürliche Selektionsprozesse waren, verliefen Resistenzentwicklungen sehr langsam. Mit der Entdeckung von natürlichen Antibiotika wie Penicillin und der Entwicklung synthetischer Antibiotika wie Chinolone und deren intensiver Anwendung durch den Menschen bei Mensch, Tier und in der Umwelt breiten sich lang vorhandene natürliche und neu entwickelte Resistenzen dramatisch schnell aus.

Weltweit war 2023 der Erreger jeder 6. laborbestätigten bakteriellen Infektion resistent gegen therapierelevante Antibiotika nach dem Global Antimicrobial Resistance and Use Surveillance System (GLASS) der WHO [5]. Anders ausgedrückt starben im Jahr 2021 schätzungsweise 4,71 Mio. Patient:innen weltweit an einer bakteriellen Infektion mit einem resistenten Erreger (assoziierte Todesfälle). Dabei ist zu beachten, dass ein Teil auch verstorben wäre, wenn der Erreger nicht resistent gewesen wäre. Allein auf die Resistenz bei einer Infektion mit einem resistenten Erreger zurückzuführen sind 1,14 Mio. (zuschreibbare/attributable) Todesfälle. Diese Zahlen werden voraussichtlich bis 2050 auf 8,22 Mio. bzw. 1,91 Mio. ansteigen [6]. Zum Vergleich: Die Anzahl der weltweiten Todesfälle durch COVID-19 im Jahr 2021 betrug laut der Global-Burden-of-Disease-Studie 7,88 Mio. [7].

In Deutschland lag die Anzahl der assoziierten Todesfälle durch Infektionen mit antibiotikaresistenten Erregern im Jahr 2019 bei ca. 45.700 und der attributablen Todesfälle bei ca. 9600 [8]. Damit starben im Jahr 2019 in Deutschland 3‑mal so viele Menschen an zuschreibbaren Todesfällen durch Infektionen mit antibiotikaresistenten Erregern wie bei Verkehrsunfällen. Anders ausgedrückt: Jede Stunde kam eine Person infolge von Antibiotikaresistenzen ums Leben. Damit sind Todesfälle durch antibiotikaresistente Erreger eine der häufigsten infektiologischen Todesursachen in Deutschland. Zum Vergleich: Nach Angaben des Statistischen Bundesamtes starben im Jahr 2021 ca. 71.300 Patient:innen in Deutschland an COVID-19 [27].

## Datengrundlage

Die Antibiotika-Resistenz-Surveillance (ARS) ist ein laborbasiertes, freiwilliges Surveillance-System des Robert Koch-Instituts [9]. Neben der nationalen Surveillance tragen ARS-Surveillance-Daten aus Deutschland zum Europäischen Netzwerk zur Überwachung von Antibiotikaresistenzen (EARS-Net) und zu GLASS bei. Die aktuellsten Daten des Überwachungssystems werden in aggregierter Form über interaktive Berichte auf der Projektwebsite bereitgestellt [28]. Im Jahr 2024 übermittelten 104 Labore Daten aus über 750 Allgemeinkrankenhäusern, dies entspricht über 50 % der Akutkrankenhäuser, und fast 30.000 Praxen, somit fast 43 % aller Praxen.

Die teilnehmenden Labore liefern Daten zur Routinediagnostik eines breiten Spektrums klinisch relevanter Erreger. Alle Labore sind akkreditiert und werten vornehmlich nach den Vorgaben des European Committee on Antimicrobial Susceptibility Testing (EUCAST) aus. In den Auswertungen werden Erreger sowohl bei der Angabe „sensibel“, d. h. empfindlich bei Standarddosierung (s), als auch bei „intermediär“, d. h. empfindlich bei höheren Therapiedosen (i), als sensibel gegenüber dem Antibiotikum gewertet und als resistent bei hoher Wahrscheinlichkeit eines Therapiefehlers (r). In diesem Beitrag werden Resistenzdaten zu ausgewählten Erreger-Wirkstoff-Kombinationen (Bug-Drug-Kombinationen) für* E. coli, K. pneumoniae, S. aureus, Streptococcus (S.) pneumoniae, E. faecium, P. aeruginosa *und *Acinetobacter *spp. von 518 kontinuierlich teilnehmenden Krankenhäusern und 15.212 kontinuierlich teilnehmenden Praxen aus den Jahren 2020 bis 2024 vorgestellt (Tab. S1 im Onlinematerial). Resistenzdaten werden für die einzelnen Erreger stratifiziert nach der Materialart (alle klinischen Materialien, respiratorisches Material, Urin, Material aus Wunden und Blut) und jeweils für den stationären Sektor und den ambulanten Sektor dargestellt. Ein Erreger wird gegenüber einer Antibiotikagruppe als resistent gewertet, wenn er gegenüber eines der zugehörigen Antibiotika resistent ist.

Die zweite Datenquelle sind die EARS-Net-Daten aus dem Jahr 2024 [10]. EARS-Net ist das größte öffentlich finanzierte System zur Überwachung von Antibiotikaresistenzen in Europa, erfasst Daten aus allen 30 EU- und EWR-Staaten und wird vom European Centre for Disease Prevention and Control (ECDC) koordiniert. Es werden Erstisolate aus Blutkultur und Liquor von 8 bakteriellen Krankheitserregern erfasst – *E. coli, K. pneumoniae, P. aeruginosa, Acinetobacter *spp., *S. pneumoniae, S. aureus, E. faecalis, E. faecium*. Zu jedem Erreger werden verschiedene Erreger-Wirkstoff-Kombinationen ausgewertet und in einer interaktiven Datenbank zur Verfügung gestellt [10, 29].

Die EARS-Net-Daten von Deutschland kommen aus ARS. Hierbei gibt es leichte Abweichungen in der Methodik im Vergleich zu den ARS-Daten, beispielsweise bei der Definition der kontinuierlichen Teilnahme über Labore oder Krankenhäuser oder den Zeitpunkt der Validierung. Dadurch können sich leichte Unterschiede bei den Ergebnissen ergeben.

**Abb. 1:**
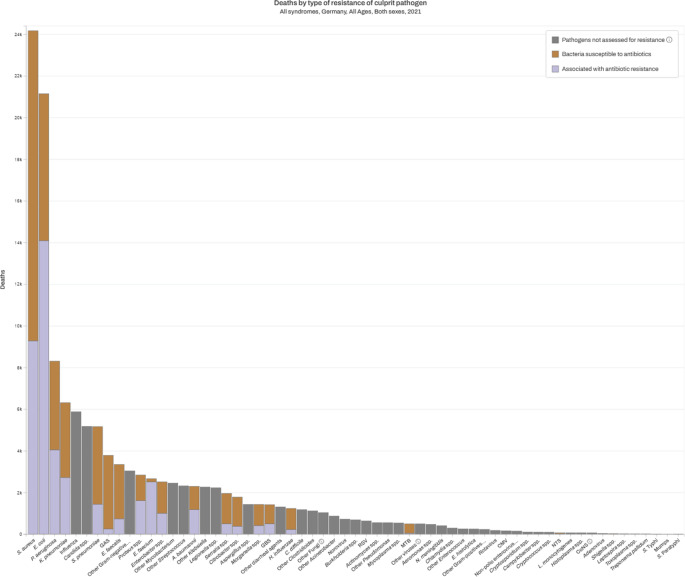
Daten des Projekts MICROBE (Measuring Infectious Causes and Resistance Outcomes for Burden Estimation). Anzahl der Todesfälle pro Erreger durch alle Infektionen in Deutschland im Jahr 2021. *Lila*: Anteil der Todesfälle, der mit Antibiotikaresistenz assoziiert ist (22 Erreger). Bildquelle: [26]

Zusätzlich werden Daten des MICROBE-Projektes (Measuring Infectious Causes and Resistance Outcomes for Burden Estimation), einer Kooperation des Institute for Health Metrics and Evaluation (IHME) und der University of Oxford, vorgestellt [6, 11]. In MICROBE werden 62 Krankheitserreger (Bakterien, Viren, Parasiten und andere Erreger, Abb. 1) analysiert, davon 22 bakterielle Erreger mit 84 Erreger-Wirkstoff-Kombinationen und 22 Infektionssyndromen in 204 Ländern und Gebieten im Zeitraum von 1990 bis 2021. Dabei werden Daten zu Todesursachen, Krankenhausentlassungen, Mikrobiologie, Literaturstudien, Arzneimittelverkäufen, Antibiotikaverbrauchsstudien, Mortalitätsüberwachung, Daten zu ambulanten und stationären Versicherungsleistungen sowie publizierte Studien ausgewertet.

## Ergebnisse

### ARS-Daten

Die Anzahl der nachgewiesenen Isolate der 7 in diese Auswertungen eingeschlossenen Erreger stieg in den von 2020 bis 2024 kontinuierlich teilnehmenden Einrichtungen bei allen Erregern bis auf *Acinetobacter (A.) baumannii* complex an (Tab. 1).

**Tab. 1:** Daten der Antibiotika-Resistenz-Surveillance (*ARS*): Anzahl der nachgewiesenen Erreger von 2020 bis 2024 von 518 kontinuierlich teilnehmenden Krankenhäusern. Alle Materialien ohne Screening, Erstisolat pro Patient:in pro Erreger pro Quartal.

	2020	2021	2022	2023	2024	Prozentuale Veränderung 2024 gegenüber 2020
*Escherichia coli*	547.618	564.528	565.440	595.399	589.158	7,6
*Klebsiella pneumoniae*	107.309	109.251	110.561	119.750	124.080	15,6
*Acinetobacter baumannii *complex	10.261	9371	8874	10.047	9750	−5,0
*Pseudomonas aeruginosa*	93.178	93.582	91.666	98.384	100.873	8,3
*Enterococcus faecium*	2904	3352	3065	3104	3167	9,1
*Staphylococcus aureus*	204.029	194.969	191.760	203.240	208.531	2,2
*Streptococcus pneumoniae*	10.760	10.704	14.059	19.091	31.525	193,0

**Abb. 2:**
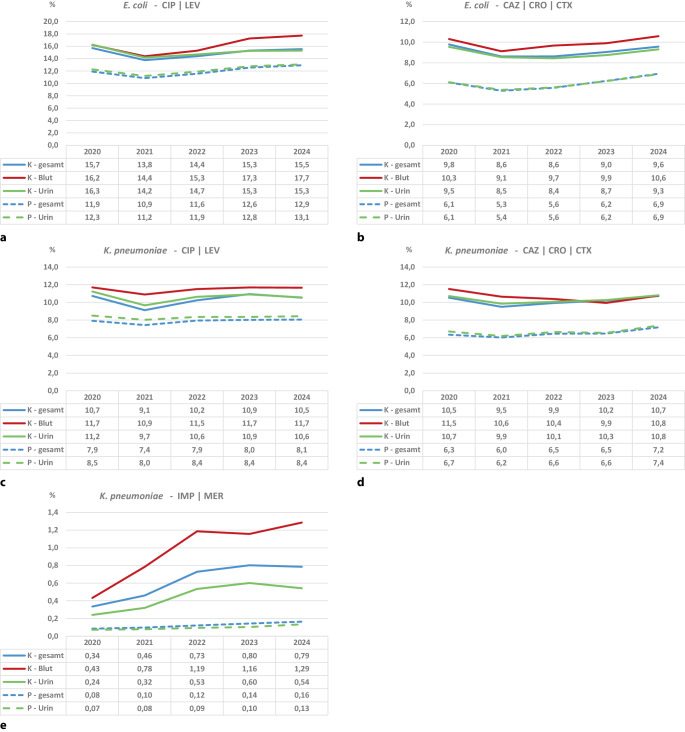
Daten der Antibiotika-Resistenz-Surveillance (*ARS*): Darstellung der Resistenzanteile von *Escherichia* *(E.) coli *und *Klebsiella* *(K.) pneumoniae* in Blutstrominfektionen in den Jahren 2020 bis 2024 von 518 kontinuierlich teilnehmenden Krankenhäusern. **a** Resistenzanteile von *E.* *coli* gegenüber Fluorchinolonen. **b** Resistenzanteile von *E.* *coli* gegenüber Cephalosporinen der 3. Generation. **c** Resistenzanteile von *K.* *pneumoniae* gegenüber Fluorchinolonen. **d** Resistenzanteile von *K.* *pneumoniae* gegenüber Cephalosporinen der 3. Generation. **e** Resistenzanteile von *K.* *pneumoniae* gegenüber Carbapenemen. *CIP* Ciprofloxacin, *LEV* Levofloxacin, *CAZ* Ceftazidim, *CRO* Ceftriaxon, *CTX* Cefotaxim, *IMP* Imipenem, *MER* Meropenem, *gesamt* alle Materialien, *K* Krankenhaus, *P* Praxis

Während im Zeitraum die Resistenzanteile von *E. coli* gegenüber Fluorchinolonen (Ciprofloxacin, Levofloxacin) für alle Materialien bzw. Urin im stationären Bereich von 15,7 % bzw. 16,3 % auf 15,5 % bzw. 15,3 % sanken, stiegen sie im Blut von 16,2 % auf 17,7 % an (Abb. 2a). Im ambulanten Bereich stiegen die entsprechenden Anteile für alle Materialien bzw. im Urin von 11,9 % auf 12,9 % bzw. 12,3 % auf 13,1 % an. Diese Entwicklungen resultierten aus einem deutlichen Abfall im Jahr 2021 und einem darauffolgenden unterschiedlich starken Anstieg über die Jahre 2022 bis 2024.

Ein ähnliches Bild zeigt sich bei den Resistenzanteilen von *E. coli* gegenüber Cephalosporinen der 3. Generation (Ceftazidim, Ceftriaxon, Cefotaxim): Auch hier ist ein Anstieg im Blut und im ambulanten Bereich zu beobachten, während im stationären Bereich in allen Materialien sowie im Urin ein leichter Rückgang erkennbar ist. Der Resistenzanteil für *E. coli* und Cephalosporinen der 3. Generation ist über alle Jahre verglichen am höchsten im Blut und lag 2024 bei 10,6 % (Abb. 2b). Die Resistenzanteile im ambulanten Bereich lagen über die Jahre ca. 3,5 Prozentpunkte unter denen im stationären Bereich. Resistenzanteile von *E. coli* gegenüber Carbapenemen sind nicht dargestellt, da diese durchgehend deutlich unter 1 % lagen.

Auch bei *K. pneumoniae* sind die höchsten Resistenzanteile bei Isolaten aus Blutkulturen zu sehen. Für Fluorchinolone wurden die niedrigsten Werte auch im Jahr 2021 ermittelt. Die Verläufe sind ähnlich wie bei *E. coli,* nur ca. 5 Prozentpunkte niedriger (Abb. 2c). Dagegen liegen die Resistenzanteile von *K. pneumoniae* gegenüber Cephalosporinen der 3. Generation leicht über denen von *E. coli*. Hier ist vor allem im ambulanten Bereich ein leichter Anstieg zu beobachten (Abb. 2d). Bei den Resistenzanteilen gegenüber Carbapenemen (Imipenem, Meropenem) ist im stationären Bereich auf niedrigem Niveau ein deutlicher Anstieg zu sehen, sodass der Resistenzanteil im Blut im Jahr 2024 bei 1,29 % liegt (Abb. 2e).

**Abb. 3:**
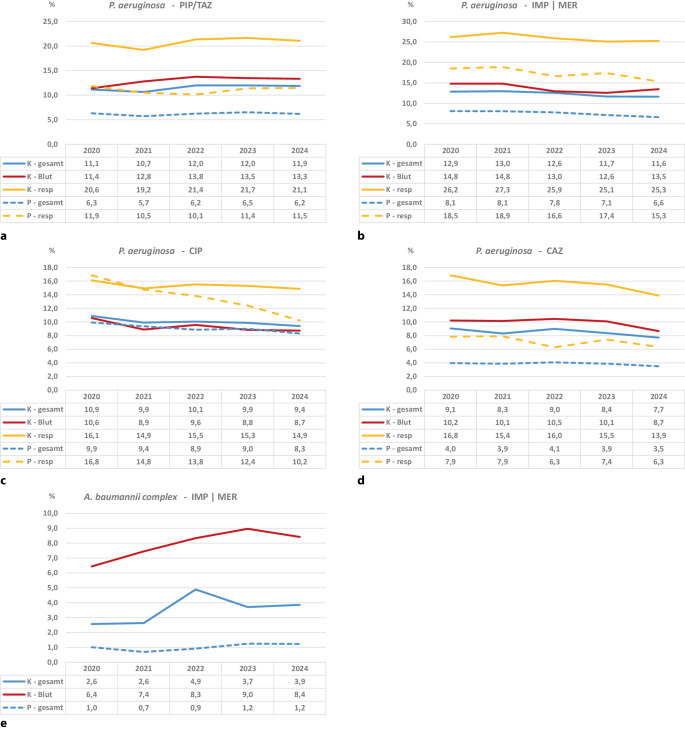
Daten der Antibiotika-Resistenz-Surveillance (*ARS*): Darstellung der Resistenzanteile von *Pseudomonas* *(P.) aeruginosa* und *Acinetobacter* *(A.) baumannii *complex in Blutstrominfektionen in den Jahren 2020 bis 2024 von 518 kontinuierlich teilnehmenden Krankenhäusern. **a** Resistenzanteile von *P.* *aeruginosa* gegenüber Piperacillin (*PIP*)/Tazobactam (*TAZ*). **b** Resistenzanteile von *P.* *aeruginosa* gegenüber Carbapenemen. **c** Resistenzanteile von *P.* *aeruginosa* gegenüber Ciprofloxacin (*CIP*). **d** Resistenzanteile von *P.* *aeruginosa* gegenüber Ceftazidim (*CAZ*). **e** Resistenzanteile von *A.* *baumannii *complex gegenüber Carbapenemen. *LEV* Levofloxacin, *CRO* Ceftriaxon, *CTX* Cefotaxim, *IMP* Imipenem, *MER* Meropenem, *gesamt* alle Materialien, *resp* respiratorische Materialien, *K* Krankenhaus, *P* Praxis

Die Resistenzanteile von *P. aeruginosa* gegenüber Piperacillin/Tazobactam haben sich über die Zeit wenig verändert, mit leicht höheren Werten im Jahr 2024 im Vergleich zu 2020 (Abb. 3a). Die Resistenzanteile im respiratorischen Material sind im stationären und im ambulanten Bereich mit 21,1 % und 11,5 % am höchsten, wobei für den stationären Bereich die Resistenzanteile in den respiratorischen Materialien fast doppelt so hoch wie in Blut und allen Materialien sind. Gegenüber Carbapenemen war der Resistenzanteil im stationären und ambulanten Bereich ebenfalls etwa 2‑mal so hoch im respiratorischen Material im Vergleich zu Blut und allen Materialien mit Anteilen von 25,3 % und 15,3 % im Jahr 2024 (Abb. 2b). Außer für die Resistenzanteile für Piperacillin/Tazobactam sanken in allen weiteren untersuchten Kombinationen und über alle Materialien hinweg die Resistenzanteile über die Zeit (Abb. 3b–d). Gegenüber Ciprofloxacin sanken die Resistenzanteile von *P. aeruginosa* besonders stark im respiratorischen Material im ambulanten Bereich (von 16,8 % auf 10,2 %). Auch gegenüber Ceftazidim sanken alle untersuchten Strata (Abb. 3d).

Die Resistenzanteile von *A. baumanii* complex gegenüber Carbapenemen stiegen an und lagen im Blut bei 8,4 % im Jahr 2024 (Abb. 3e).

**Abb. 4:**
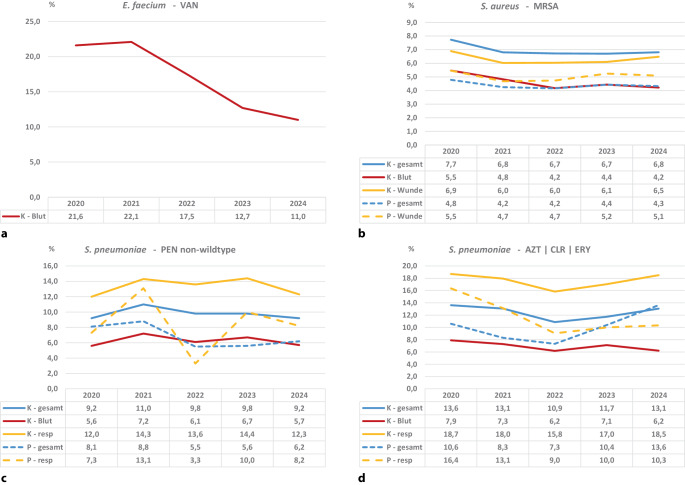
Daten der Antibiotika-Resistenz-Surveillance (*ARS*): Darstellung der Resistenzanteile von *Staphylococcus* *(S.) aureus*, *Enterococcus* *(E.) faecium* und *Streptococcus* *(S.) pneumoniae* in Blutstrominfektionen in den Jahren 2020 bis 2024 von 518 kontinuierlich teilnehmenden Krankenhäusern. **a** Anteile von Methicillin-resistenten *S.* *aureus *(*MRSA*). **b** Anteile von Vancomycin-resistenten *E.* *faecium *(*VRE*). **c** Resistenzanteile von *S.* *pneumoniae* gegenüber Penicillin (*PEN*). **d** Resistenzanteile von *S.* *pneumoniae* gegenüber Makroliden. *AZM* Azithromycin, *CLR* Clarithromycin, *ERY* Erythromycin, *resp* respiratorische Materialien, *K* Krankenhaus, *P* Praxis

Der Anteil von MRSA an allen *S. aureus* ist in allen untersuchten Strata im Jahr 2024 im Vergleich zum Ausgangswert von 2020 gesunken. Im Blut liegt der Anteil im Jahr 2024 bei 4,2 %, in Wunden bei 6,5 % im stationären Bereich und 5,1 % im ambulanten Bereich (Abb. 4a). Der Anteil VRE an allen *E. faecium* wird nur für Blut untersucht und sank im Zeitraum von 21,6 % auf 11,0 % (Abb. 4b).

Die Anteile von *S. pneumoniae* mit Resistenz gegenüber Penicillin (Nicht-Wildtyp, d. h. Bakterium mit Mutation im Genom) liegen um 10 % bei respiratorischem Material und sind damit fast doppelt hoch wie im Blut (2020: 5,6 % und 2024: 5,7 %; Abb. 4c). Auch bei den Resistenzanteilen gegenüber Makroliden liegen die Werte im Blut deutlich unter denen im respiratorischen Material (2024: 6,2 % und 18,5 %; Abb. 4d).

### EARS-Net-Daten

Zur nationalen Einschätzung der Antibiotikaresistenz ist ein Vergleich mit anderen Ländern wichtig. Die EU hat für alle Staaten Ziele vorgegeben. Jährlich berechnet das ECDC anhand der übermittelten Surveillance-Daten den Fortschritt der Länder. Als Maßzahl werden Inzidenzen von Blutstrominfektionen berechnet, da dies eine bessere Vergleichbarkeit mit anderen Krankheiten ermöglicht und hier neben dem Resistenzanteil auch die absolute Anzahl der Erreger in das Ergebnis eingeht.

**Abb. 5:**
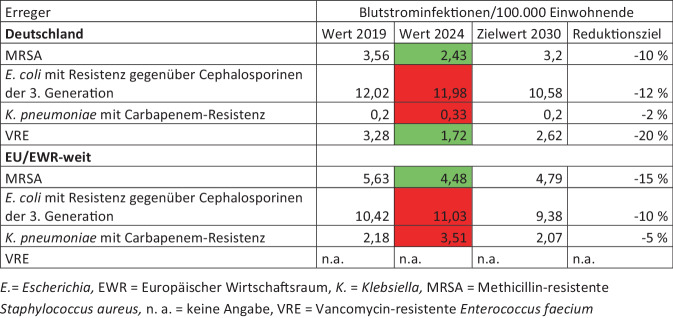
Daten aus dem Europäischen Netzwerk zur Überwachung von Antibiotikaresistenzen (EARS-Net; [10]): Reduktionsziele der EU und aus dem 1. Aktionsplan der Deutschen Antibiotika-Resistenzstrategie (DART) 2030. *Grün* = Zielwert 2030 erreicht. *Rot* = Zielwert 2030 noch nicht erreicht

In der EU sind sowohl die Inzidenz der Blutstrominfektionen von 3.-Generation-Cephalosporin-resistenten *E. coli* als auch von Carbapenem-resistenten *K. pneumoniae* seit 2019 angestiegen. In Deutschland ist die Inzidenz für 3.-Generation-Cephalosporin-resistente *E. coli* zwar gegenüber 2019 leicht reduziert, 2024 aber das dritte Jahr in der Folge angestiegen. Für MRSA sind sowohl die EU-Inzidenz als auch die Inzidenz für Deutschland gesunken und bereits unterhalb der Zielwerte für das Jahr 2030 (Abb. 5).

Im EARS-Net-Jahresbericht 2024 wird der Stand aller Länder gesondert dargestellt [10]. Bei der Inzidenz der 3.-Generation-Cephalosporin-resistenten *E. coli* liegt Deutschland an Position 24 der 30 EU-/EWR-Länder, bei Carbapenem-resistenten *K. pneumoniae* an Position 13 und bei MRSA an Position 12. Einige Länder wie Frankreich und Finnland haben bereits jetzt eine größere Reduktion erreicht als für 2030 angezielt.

### IHME-/MICROBE-Daten

Die Erreger, die im Gesundheitswesen häufig vorkommen und wegen ihrer Resistenz schwierig zu behandeln sind, stellen die größte Krankheitslast dar. Mit Abstand sind *S. aureus* und *E. coli* für die meisten Todesfälle verantwortlich (Abb. 1), es folgen *P. aeruginosa* und *K. pneumoniae*. Diese 4 Erreger sind für über 50.000 Todesfälle im Jahr 2021 in Deutschland verantwortlich. Bei *E. coli* sind deutlich über 50 % der Todesfälle mit Antibiotikaresistenz assoziiert (Abb. 1). Erst an 5. Stelle kommt mit Influenza ein Virus. Über 90.000 Todesfälle sind auf bakterielle Infektionen zurückzuführen. Über 40.000 sind mit Antibiotikaresistenz assoziiert und über 8000 sind allein auf die Resistenz zurückzuführen (attributabel). Der Rückgang gegenüber 2019 ist hauptsächlich auf eine neue Art der Berechnung und den Rückgang von Infektionen mit MRSA zurückzuführen.

Neben den Daten des MICROBE-Projekts hat eine weitere Studie (Cassini et al.) die Anzahl der Todesfälle für eine Auswahl von resistenten Erregern für das Jahr 2015 auch für Deutschland berechnet [12]. Auch Cassini stellt die durch Antibiotikaresistenzen verursachte Krankheitslast der Belastung durch andere Infektionskrankheiten gegenüber und berechnet sie vergleichbar mit der kumulativen Krankheitslast durch Influenza, Tuberkulose und HIV in der EU und dem EWR im Jahr 2015. Beide Studien kommen mit unterschiedlichen Methoden der Krankheitslastberechnung (Inzidenzansatz und Prävalenzansatz) zu sehr ähnlichen Ergebnissen, was Meštrović et al. zeigen konnten [8].

## Diskussion

Die Entwicklungen der Resistenzsituation sind teilweise kritisch. Das Bild ist aber im Detail uneinheitlich. Ein Grund dafür ist die COVID-19-Pandemie, die im Beobachtungszeitraum von 2020 bis 2024 lag [13]. Die Resistenzanteile unterscheiden sich nach Material und Sektor, höhere Werte sind im stationären Bereich und insbesondere bei *P. aeruginosa* in respiratorischen Materialien zu beobachten. Einzelne Erreger-Wirkstoff-Kombinationen zeigen sogar gegensätzliche Trends im ambulanten und stationären Bereich. Bei den grampositiven Erregern sind hauptsächlich rückläufige Trends zu erkennen. Während dies für MRSA schon seit 15 Jahren der Fall ist, fallen die Zahlen bei VRE seit 2021 deutlich [14].

Bei den gramnegativen Erregern ist die Situation kritischer. Schwierig einzuordnen ist die Fluorchinolon-Resistenz. Der erwartete Rückgang aufgrund der Reduktion der Fluorchinolon-Gabe ist nur bei *P. aeruginosa* im ambulanten Bereich deutlich zu sehen, hingegen steigen die Resistenzen bei *E. coli* im ambulanten Bereich sogar an.

Sowohl bei *K. pneumoniae* als auch beim *A. baumanii* complex sind Anstiege der Resistenzen gegenüber Carbapenemen zu sehen. Hier wirken nur noch Reserveantibiotika und es droht in Deutschland eine gefährliche Entwicklung, wie z. B. in Italien, wo in den Jahren 2009 bis 2011 ein Anstieg der Resistenz von *K. pneumoniae* gegenüber Carbapenemen von 1,3 % auf über 26 % zu beobachten war [15, 16]. Neben Antibiotic Stewardship und Infektionskontrolle ist es daher wichtig, Strukturen wie die Integrierte Genomische Surveillance (IGS) zu stärken, die eine Nachverfolgbarkeit von resistenten Erregern bis zu einzelnen Resistenzgenen und eine Verkürzung von Ausbrüchen ermöglicht [17, 18].

Diese kritische Situation zeigt sich auch bei den Reduktionszielen der DART 2030. Es ist gut, dass solche Ziele existieren. Sie fördern das kollektive Bewusstsein, gemeinsam an einer Sache zu wirken. Bei Carbapenem-resistenten *K. pneumoniae* sieht die Situation jedoch schlecht aus, im 4. Jahr in Folge steigt die Inzidenz von Blutstrominfektionen an. Nur ein etwas besseres Bild zeigt sich bei Blutstrominfektionen von 3.-Generation-Cephalosporin-resistenten *E. coli*. Hier steigt die Inzidenz seit 3 Jahren an.

Interessant ist, dass Deutschland beim Resistenzanteil deutlich unterhalb des EU-Durchschnitts liegt, bei Betrachtung der Inzidenz allerdings deutlich darüber. Grund dafür ist der sehr häufige Nachweis von *E. coli* in Deutschland. In die Inzidenz geht auch die Anzahl der Erreger ein, daher kann mit diesem Parameter die Belastung des Gesundheitswesens besser beurteilt werden. Die Verbreitung der Reduktionsziele und die Umsetzung von Maßnahmen, um diese zu erreichen, müssen Hauptaufgaben in allen Instanzen öffentlicher Gesundheit sowie im Bereich der Patientenversorgung (insbesondere in der Krankenhaushygiene), von Antibiotic Stewardship und letztendlich der gesamten Gesundheitsversorgung sein.

Der Anstieg der Erregerzahlen über die Untersuchungszeit lässt sich teilweise durch die COVID-19-Pandemie erklären, während der die Zahl der Patient:innen sehr niedrig war und danach wieder angestiegen ist. Der deutliche Anstieg von *S. pneumoniae* mit fast 300 % ist am eindeutigsten auf die Pandemie zurückzuführen [19].

Die Daten von MICROBE zeigen den Stellenwert, den Antibiotikaresistenz in Deutschland hat. Allein aufgrund der Resistenz sterben fast 10.000 Menschen im Jahr an einer bakteriellen Infektion, dies sind deutlich mehr als durch andere Infektionskrankheiten.

### Syndemie der antimikrobiellen Resistenz

Viele Faktoren haben Einfluss auf die Antibiotikaresistenz von bakteriellen Erregern. Auch bei der Antibiotikaresistenz spielen soziale Faktoren eine Rolle. So konnten Singer et al. zeigen, dass es einen deutlichen Zusammenhang zwischen sozioökonomischem Status und MRSA in Deutschland gibt [20, 21]. In Landkreisen mit niedrigerer sozioökonomischer Position werden häufiger invasive Infektionen mit MRSA nachgewiesen.

Der enge Zusammenhang von Antibiotikaresistenz mit anderen insbesondere nichtübertragbaren Krankheiten ist herausfordernd. Es existieren zunehmend Autor:innen, die einer syndemischen Perspektive, d. h. der sich gegenseitig verstärkenden Verflechtung von Risikofaktoren und Komorbiditäten, mehr Aufmerksamkeit widmen [20, 21].

Über soziale Determinanten und pathophysiologische Mechanismen hängen Antibiotikaresistenz und nichtübertragbare Krankheiten oft zusammen. Eine besondere Rolle spielen onkologische und hämatologische Erkrankungen. Im Jahr 2022 veröffentlichten Global Health Dynamics und die Union for International Cancer Control Artikel zu den Herausforderungen, die Antibiotikaresistenzen für die Krebsgemeinschaft darstellen [22]. In einem Scoping-Review wurde festgestellt, dass 35 % der Patient:innen mit hämatologischen Erkrankungen eine Infektionen mit kritischen resistenten Erregern der Prioritätenliste der WHO haben [23, 24].

Antibiotikaresistenz hat nicht nur negative Auswirkungen auf hämatoonkologische Erkrankungen, sondern auch auf invasive Maßnahmen wie Operationen. Perioperative Prophylaxe muss zunehmend mit Breitspektrum Antibiotika durchgeführt werden, die Behandlung postoperativer Infektionen wird noch schwieriger [25].

Ein weiterer deutlicher Faktor für die Notwendigkeit der syndemischen Perspektive ist die starke inverse Korrelation zwischen Einkommensniveau und Gesundheitsversorgung einerseits und dem Anteil resistenter Erreger an Blutkulturinfektionen andererseits auf globalem Niveau [5].

## Fazit

Das Thema Antibiotikaresistenz wird in der Krankenversorgung in den nächsten Jahren eine immer größere Rolle einnehmen. Während viele andere Infektionskrankheiten durch Maßnahmen zurückgedrängt werden können und teilweise berechtigte Ziele der Eradikation existieren, muss davon ausgegangen werden, dass Infektionen mit resistenten Erregern zunehmen werden. Dank moderner Medizin können Patient:innen trotz eingeschränkter Immunlage länger behandelt werden – hierfür ist jedoch häufig der Einsatz von Antibiotika notwendig. Im globalen Süden entwickeln sich die Gesundheitssysteme weiter, wodurch die Menschen länger leben und häufiger an sogenannten Zivilisationskrankheiten erkranken. Dadurch wird in den kommenden Jahren ein höherer Verbrauch an Antibiotika entstehen, was voraussichtlich zu einem weiteren Anstieg der Resistenzen führen wird. In diesem Sinne korreliert das Auftreten von Infektionen mit resistenten Erregern perspektivisch eher mit dem Anstieg nichtübertragbarer Erkrankungen als mit Infektionskrankheiten.

Nur durch konsequente Umsetzung von Maßnahmen wie Antibiotic Stewardship, Krankenhaushygiene und Infektionskontrolle sowie Impfungen kann die deutliche Zunahme von Infektionen mit resistenten Erregern eingedämmt werden.

## Supplementary Information

ESM1: Zusatzmaterial 1

## Data Availability

Ein Teil der Daten ist im Manuskript enthalten, weitere in dieser Studie erhobenen Datensätze können auf begründete Anfrage beim Korrespondenzautor angefordert werden.
