# Supplementary material for: Development of the epidemiology of antibiotic-resistant pathogens in human medicine and their significance for the health system in Germany
Source: Bundesgesundheitsblatt Gesundheitsforschung Gesundheitsschutz. 2026 Apr 27;69(5):546–55. [Article in German] doi: 10.1007/s00103-026-04234-6 (PMC13132887; doi:10.1007/s00103-026-04234-6)
Supplement: Supplementary file 1 — ESM1: Zusatzmaterial 1 [file 103_2026_4234_MOESM1_ESM.docx]

Anhang

**Entwicklung der Epidemiologie antibiotikaresistenter Erreger in der Humanmedizin und deren Bedeutung für das Gesundheitssystem**

Development of the epidemiology of antibiotic-resistant pathogens in human medicine and their significance for the health system, in Germany

Tim Eckmanns, Felix Reichert, Marc Schneider, Marcel Feig, Ines Noll, Alexandra Hoffmann

Robert Koch-Institut, Abteilung für Infektionsepidemiologie

Tab. S1: Erreger-Wirkstoff-Kombinationen, die mit Daten der Antibiotika-Resistenz-Surveillance (ARS) ausgewertet wurden.

CIP: Ciprofloxacin, LEV: Levofloxacin, CAZ: Ceftazidim, CRO: Ceftriaxon, CTX: Cefotaxim, IMP: Imipenem, MER: Meropenem, PIP/TAZ: Piperacillin-Tazobactam, MRSA: Methicillin-resistenter *S. aureus*, VRE: Vancomycin resistenter *Enterococcus faecium*, PEN: Penicillin, AZM: Azithromycin, CLR: Clarithromycin, ERY: Erythromycin
